# Supplementary material for: Determination of Free Amino Acids in Milk, Colostrum and Plasma of Swine via Liquid Chromatography with Fluorescence and UV Detection
Source: Molecules. 2022 Jun 28;27(13):4153. doi: 10.3390/molecules27134153 (PMC9268350; doi:10.3390/molecules27134153)
Supplement: Supplementary file 1 [file molecules-27-04153-s001.zip › molecules-1771522-supplementary.pdf]

Supplementary material

All results were obtained by applying chromatographic method 1, except proline analyzed with method 2.

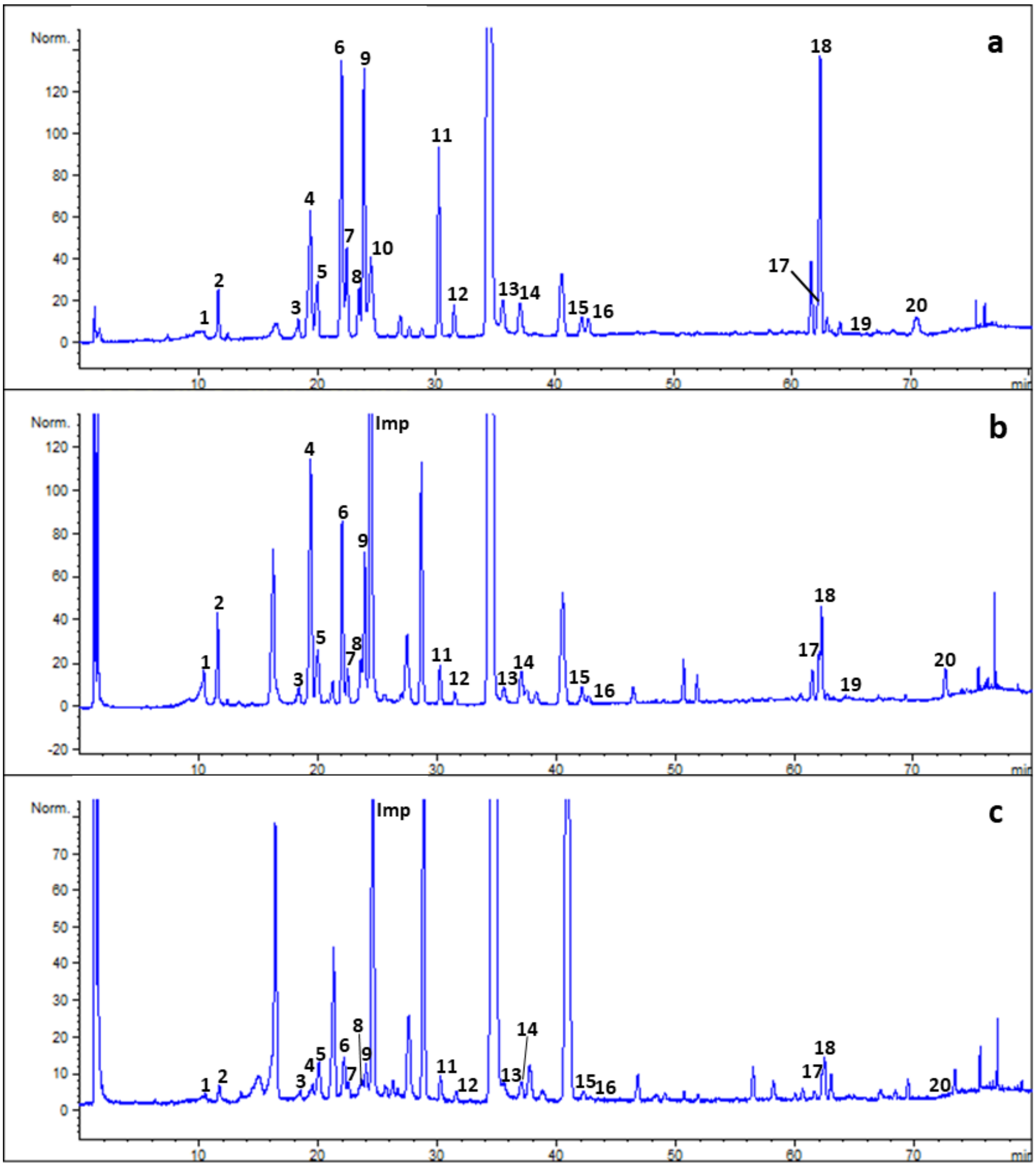

Figure S 1 Chromatograms of sample solutions: a) Plasma, b) Milk, c) Colostrum. FAAs: 1-Asp, 2-Glu, 3-Asn, 4-Gln, 5-Ser, 6-Gly, 7-Thr, 8-Arg, 9-Ala, 10-Pro, 11-Val, 12-Met, 13-Ile, 14-Leu, 15-Phe, 16-Trp, 17-His, 18-Lys, 19-Cys, 20-Tyr. Method 1, mobile mobile phase A: 40 mM aqueous NaH_2PO_4 buffer, pH 8.5, B: CH_3CN / CH_3OH / H_2O / H_3PO_4 45:45:10:0.1 (v/v); gradient 1; Kinetex F5 column. Detection: DAD at 216 nm.

Table S1. Matrix effect (ME%) values in plasma, colostrum and milk.

	ME% in plasma			ME% in milk			ME% in colostrum		
	0.07 µg/mL	0.7 µg/mL	14.0 µg/mL	0.07 µg/mL	0.7 µg/mL	14.0 µg/mL	0.07 µg/mL	0.7 µg/mL	14.0 µg/mL
Asp	-3.1	-4.3	-3.8	-7.9	-6.6	-5.0	-5.2	-10.2	-9.8
Glu	+1.2	-1.2	-0.6	-1.5	-8.1	-7.1	-1.2	-7.2	-8.6
Asn	-4.7	-4.8	-3.4	-4.3	-6.9	-6.4	-6.7	-7.8	-8.4
Gln	-2.8	+1.3	-2.5	-10.5	-8.7	-9.4	-11.8	-12.3	-7.5
Ser	-2.2	-3.5	-3.5	-7.2	-7.1	-8.0	-8.2	-6.5	-8.5
Gly	-3.2	-2.6	-3.1	-10.4	-11.0	-9.4	-8.6	-8.1	-11.5
Thr	+1.5	+1.1	-1.3	-9.5	-9.9	-10.7	-6.8	-13.5	-10.3
Arg	+0.9	+2.4	+3.3	5.6	8.8	6.1	-8.4	-6.6	-12.4
Ala	-2.5	-4.1	-4.4	-10.1	-10.7	-10.5	-9.2	-8.4	-7.2
Pro	-5.2	-4.9	-4.1	-10.3	-7.7	-6.9	-11.2	-10.5	-10.2
Val	+2.1	+1.0	-0.3	-5.1	+2.3	-1.9	+2.3	-4.4	-7.4
Met	-3.0	-2.3	-2.7	-5.8	-7.0	-6.1	-6.4	-7.4	-5.3
Ile	-5.5	-4.2	-4.1	-11.3	-8.7	-8.3	-5.3	-12.4	-6.2
Leu	-2.5	-2.1	-2.8	-1.4	-4.5	-3.2	-3.2	-5.5	-7.1
Phe	-4.2	-3.3	-2.9	-8.3	-7.7	-7.9	-6.2	-8.4	-10.1
Trp	+2.6	-1.8	+1.3	-7.1	-6.6	-5.9	-8.6	-8.2	-5.8
His	-9.9	-6.9	-8.3	-8.7	-7.7	-6.2	-11.3	-10.2	-4.5
Lys	+5.6	+2.2	+3.8	-3.8	-4.9	-2.7	-4.2	-11.2	-6.6
Cys	-10.4	-12.1	-9.8	-11.1	-12.5	-15.1	-13.2	-12.9	-10.8
Tyr	+0.5	+1.3	-1.8	-4.4	-3.7	-2.5	-4.9	-3.4	-4.3

Table S2. Linearity results for calibration of the 20 amino acids (concentrations 0.07, 0.30, 0.7, 7.0, 14.0, 60.0 µg/mL).

	Equation	Determination coefficient (r^2)
Asp	y = 30117x - 6.5478	0.9936
Glu	y = 30586x - 17.582	0.9920
Asn	y = 45457x - 13.264	0.9915
Gln	y = 37762x - 8.3316	0.9983
Ser	y = 56035x + 14.435	0.9979
Gly	y = 74308x - 18.189	0.9941
Thr	y = 37149x + 0.3176	0.9961
Arg	y = 25019x + 3.1782	0.9993
Ala	y = 55741x + 4.9738	0.9985
Pro	y = 37055x - 3.12	0.9910
Val	y = 50566x - 14.571	0.9966
Met	y = 39027x - 13.606	0.9966
Ile	y = 39053x - 1.0455	0.9969
Leu	y = 40133x - 7.7901	0.9976
Phe	y = 28875x - 4.9142	0.9965
Trp	y = 29547x - 3.4378	0.9967
His	y = 69931x - 38.491	0.9948
Lys	y = 66849x - 42.11	0.9903
Cys	y = 70605x - 3.0829	0.9946
Tyr	y = 63553x - 9.9893	0.9971

Table S3. LOD and LOQ values of the developed method for all the investigated compounds.

	LOD (ng/mL)	LOQ (ng/mL)
Asp	6	20
Glu	15	50
Asn	6	20
Gln	6	20
Ser	5	15
Gly	3	10
Thr	6	20
Arg	4	13
Ala	5	15
Pro	15	50
Val	5	15
Met	10	30
Ile	15	50
Leu	15	50
Phe	15	50
Trp	15	50
His	3	10
Lys	5	15
Cys	4	12
Tyr	4	13

Table S4. Accuracy, determined as mean recovery percentage (Rec%) values, in plasma, colostrum and milk (spike concentration 0.07, 0.7, 14.0 µg/mL).

	Rec % from milk	Rec % from colostrum	Rec % from plasma
Asp	84,06	74,22	106,31
Glu	89,26	93,83	91,90
Asn	82,69	78,25	89,85
Gln	91,83	75,21	94,69
Ser	86,61	80,23	89,64
Gly	104,41	97,30	97,42
Thr	86,04	86,81	90,34
Arg	83,71	98,32	81,54
Ala	94,44	93,62	102,23
Pro	85,48	78.31	104,87
Val	97,65	104,45	101,17
Met	96,40	110,97	90,86
Ile	96,93	96,86	96,39
Leu	88,60	98,49	87,10
Phe	87,68	92,05	84,07
Trp	90,33	108,87	92,00
His	88,59	100,70	83,26
Lys	100,40	101,73	114,39
Cys*	70,61	70,44	71,35
Tyr	62,98	100,50	80,55

* Cys recovery on sample spiked with fresh standard solution.

Table S5. Precision values of the developed method for all the investigated compounds. Results represent the average of intra- (n=3) and inter-day (n=9) precision at the three different concentrations (0.07, 0.7, 14.0 µg/mL). Precision is expressed as coefficient of variation percent (CV%).

	Plasma		Milk		Colostrum	
	Intra-day	Inter-day	Intra-day	Inter-day	Intra-day	Inter-day
Asp	5	8	7	8	7	10
Glu	7	3	10	12	10	9
Asn	4	3	8	9	9	7
Gln	6	3	11	8	10	6
Ser	3	4	9	10	8	8
Gly	4	5	10	13	10	7
Thr	3	5	12	7	11	9
Arg	6	4	10	8	10	11
Ala	6	3	8	12	8	8
Pro	6	5	7	11	6	10
Val	5	7	11	12	11	12
Met	7	7	7	8	7	7
Ile	3	6	10	9	9	9
Leu	6	3	8	11	8	6
Phe	4	5	12	10	9	8
Trp	3	3	12	12	11	9
His	5	7	10	8	11	10
Lys	4	6	7	13	7	11
Cys	8	15	11	16	12	10
Tyr	3	4	8	10	8	9

Table S6. Concentrations of free AAs in Plasma, colostrum and milk. Results are expressed as mean concentration (n=3).

	Milk (µg/mL)[a]	Colostrum (µg/mL)[a]	Plasma (µg/mL)[a]
Asp	9,58	3,74	2,82
Glu	33,61	2,97	21,37
Asn	3,39	1,09	5,07
Gln	114,51	1,45	56,89
Ser	16,36	1,49	15,05
Gly	40,52	4,52	54,04
Thr	12,86	1,43	28,67
Arg	25,68	0,58	23,45
Ala	36,22	1,95	64,53
Pro	ND	ND	40,16
Val	12,38	3,37	57,07
Met	5,82	traces	13,80
Ile	6,63	0,80	14,70
Leu	12,34	2,37	17,88
Phe	13,80	2,01	15,84
Trp	5,79	traces	12,94
His	9,57	2,41	5,61
Lys	21,10	4,83	56,97
Cys	traces	ND	traces
Tyr	6,37	traces	9,78

[a] CV% ≤9.5.

Table S7. Analytical characteristics of recently published (2020-2022) HPLC methods for AAs analysis in milk, colostrum and plasma of livestock animals.

**Sample**	**Column/conditions**	**Type of analytes**<br>**Sample pretreatment**	**Derivatization**	**Detection**	**LOD**<br>**LOQ**<br>**µg/mL**	**ref**
Milk*	C18, gradient elution	FAAs<br>Protein precipitation ACN	OPA-MPA[1)]	UV-DAD[2)]	0.01-0.28<br>0.04-0.86	40
Cow milk	C18, gradient elution	Total AAs.<br>Lyophilization-hydrolysis<br>HCl 6M, 110°C x 24h	2,4-dinitrochlorobenzene	UV-DAD[2)]	-[3)]	41
Goat plasma	C18, gradient elution	FAAs<br>Protein precipitation ACN	OPA	FLD[4)]	-	42
Jameed (cow milk)	Ion exchange AAs analyser	Total AAs<br>Hydrolysis<br>HCl 8M, 100°C x 24h	Post-column ninhydrin	440 nm<br>570 nm	-	43
Cow/Sheep milk	Ion exchange AAs analyser	Total AAs<br>Hydrolysis HCl 7.5 M<br>Performic acid oxidation<br>Hydrolysis (NaOH 4.67M	Post-column ninhydrin	440 nm<br>570 nm	-	44
Sow serum, colostrum, milk	Ion exchange AAs analyser	FAAs<br>Protein precipitation sulfosalicylic acid	Post-column ninhydrin	440 nm<br>570 nm		30
Bovine colostrum and milk	C18, gradient elution	FAAs<br>Protein precipitation sulfosalicylic acid	iTRAQ®	HPLC-MS/MS	0.5–10 µmol/L**	38

*infant formulas and medical food
** data from J. Chromatogr. B 877 (2009) 1838-1846
1) OPA-MPA: ortho-phthalaldehyde and 3-Mercaptopropionic acid
2) UV-DAD: ultraviolet diode-array detector
3) – not reported
4) FLD: fluorescence detector
